# Yap/Taz transcriptional activity is essential for vascular regression via Ctgf expression and actin polymerization

**DOI:** 10.1371/journal.pone.0174633

**Published:** 2017-04-03

**Authors:** Ayumi Nagasawa-Masuda, Kenta Terai

**Affiliations:** Laboratory of Function and Morphology, Institute of Molecular and Cellular Biosciences, The University of Tokyo, Yayoi 1-1-1 Bunkyo-ku, Tokyo, Japan; University of Colorado Boulder, UNITED STATES

## Abstract

Vascular regression is essential to remove redundant vessels during the formation of an efficient vascular network that can transport oxygen and nutrient to every corner of the body. However, no mechanism is known to explain how major blood vessels regress during development. Here we use the dorsal part of the caudal vein plexus (dCVP) in Zebrafish to investigate the mechanism of regression and discover a new role of Yap/Taz in vascular regression. During regression, Yap/Taz is activated by blood circulation in the endothelial cells. This leads to induction of Ctgf and actin polymerization. Interference with Yap/Taz activation decreased Ctgf production, which decreased actin polymerization and vascular regression. These results implicate a novel role of Yap/Taz in vascular regression.

## Introduction

Since the vascular network is essential for oxygen and nutrient transport throughout the developing body, vascular formation is one of the most important events during early development [1–3]. Vascular formation is comprised of two main steps necessary for a functional network, new vascular formation and vascular regression. Many previous studies have focused on understanding new vascular formation, such as determining the requirement of Vegf and Notch signaling pathways for the proper sprouting of tip cells [4–7] and the requirement of Ephrin/Eph and Bmp signaling for caudal vein plexus (CVP) formation in zebrafish angiogenesis [8, 9]. In contrast, the regression process is poorly understood, especially during development. Two phenomena are defined in vascular regression: pruning and mature vessel regression [10]. Pruning has been studied with respect to cancer [11, 12] as well as in retinal angiogenesis in mice [13], and the inhibition of Vegf signaling is a key factor in the removal of juvenile vessels by pruning. A well-known mature vascular regression event is the closure of the ductus arteriosus vascular [14], which requires smooth muscle contraction and endothelial remodeling [15]. However, the mechanism of endothelial remodeling has been poorly understood.

Yes-associated protein (Yap) and its paralogous, transcriptional coactivator with PDZ-binding motif (Taz), are transcriptional factors and downstream targets of the Hippo pathway, which controls the size of organs [16–19]. The Hippo pathway is a signaling cascade activated in high cell density environments via mammalian STE20-like protein kinases 1 and 2, as well as large tumor suppressor kinase 1 and 2. The pathway suppresses cell proliferation by inhibiting the translocation of Yap/Taz to the nucleus [18, 20, 21]. This Yap/Taz translocation is also regulated by mechano-stress independently of the Hippo pathway [22] due an interaction between angiomotin and F-actin that downregulates Yap activity by retaining Yap in the cytoplasm [23]. The systematic gene knockout of Yap in mice shows lethality during early development due to defects in vascular formation in the yolk sac [24]. On the other hand, normal vasculogenesis has also been reported despite the systematic depletion of Yap in zebrafish [25]. Hence, it is controversial whether Yap/Taz transcriptional activity is an essential factor for angiogenesis during early development, and the details of Yap/Taz function are still unclear, particularly in endothelial cells (ECs).

Here, we demonstrate that Yap/Taz transcriptional activity in ECs is required for vascular regression via vascular shrinking. Inhibition of Yap/Taz transcriptional activity disturbed the regression of the dorsal part of the caudal vein plexus (dCVP), where is the first circulatory caudal vein plexus. By monitoring the activity of Yap/Taz transcription in ECs, Yap/Taz was activated during dCVP regression. Overexpression of Yap/Taz in ECs reduced the size of the dCVP, indicating that Yap/Taz is required for vessel shrinking. Furthermore, we identified a Ctgf as a responsible target of Yap/Taz during dCVP regression. The expression of Ctgf was positively regulated by Yap/Taz. The expression of exogenous Ctgf in ECs cancelled the defect of dCVP regression induced by inhibiting Yap/Taz. F-actin polymerization, one of the Ctgf dependent events, was in CVP also induced by Yap/Taz and required for dCVP regression. Our results uncover the role of Yap/Taz in ECs and provide new insights into how vascular regression is regulated.

## Materials and methods

### Zebrafish husbandry

Zebrafish (*Danio rerio*) strains were maintained under standard conditions. We used a fish medium containing 0.03% sea salt and 0.006% methylene blue as an antiseptic agent. Embryo stages were determined based on the hpf, at 28°C [26]. Microinjection and chemical treatment of embryos were undertaken as described below.

### Plasmid constructs

To prepare the plasmid for generating zebrafish lines, we constructed pTol2-fli1-gal4dbd-htead2ΔN-2A-mCherry, pTol2-UAS-EGFP-htead2ΔN, pTol2-UAS-EGFP-hyapΔC, pTol2-UAS-EGFP-hyap, pTol2-UAS-EGFP-htaz, and pTol2-UAS-zctgfa-EGFP. The Tol2 vector system was kindly provided from K. Kawakami (National Institute of Genetics, Japan) [27]. The UAS sequence was provided by M. Hibi (Nagoya University, Japan). cDNA fragments encoding human *tead2*, *yap(hyap)*, and *taz(htaz)* were amplified by PCR from cDNA libraries. cDNA of zebrafish *yap(zyap)* and *taz(ztaz)* was also amplified from cDNA libraries. cDNA for expressing and detecting *zctgfa* were kindly gifted from N. Mochizuki (National Cerebral and Cardiovascular Center research Institute, Japan) [28]. cDNA coding Ctgfa wildtype (amino acids 1–345) and N-terminal deleted mutant (amino acids 22–345) were generated by PCR and subcloned into pTol2-UAS-GFP vectors.

### Transgenic zebrafish lines

Tol2 transposase mRNA was synthesized *in vitro* with SP6 RNA polymerase from a NotI linearized pCS-TP vector. To generate the *Tg(UAS*: *EGFP-htead2ΔN)*, *Tg(UAS*: *hyapΔC-EGFP)*, *Tg(fli1*: *gal4dbd-htead2ΔN-2A-mCherry)*, *Tg(UAS*: *EGFP-hyap)*, *Tg(UAS*: *EGFP-htaz)*, and *Tg(UAS*:*zctgfa-EGFP)* zebrafish lines, the corresponding Tol2-based DNAs (100 ng/μl) were microinjected along with Tol2 transposase mRNA (25 ng/μl) into one-cell-stage embryos of the wild type strain, AB. To establish the *Tg(fli1*: *gal4dbd-htead2ΔN-2A-mCherry)* line, all injected embryos were raised to adulthood and crossed with the *Tg(UAS*: *EGFP)* line to detect the EGFP signal. To establish the other zebrafish lines, fish carrying the genes encoding the EGFP were first screened by genomic PCR, then crossed with the *Tg(fli1*: *gal4dbd-vp16)* fish line to confirm the expression of EGFP.

The *Tg(UAS*: *EGFP)* fish line was kindly provided from M. Hibi (Nagoya University, Japan) [29], and the *Tg(fli1*: *gal4dbd-vp16)* fish line was a gift from M. Affolter (University of Basel, Switzerland) [30, 31]. The *Tg(fli1*: *Myr-mCherry)* and *Tg(UAS*: *mCherry)* fish lines were provided by N. Mochizuki [32].

### Image acquisition, processing, and quantification

Zebrafish embryos were dechorionated and mounted in 1% low-melting agarose on a 35 mm glass-bottomed dish (Asahi Techno Glass) with 0.016% tricaine (Sigma-Aldrich) in fish medium, as described previously [33]. The dish was submerged in fish medium with 0.001% tricaine.

Confocal images were taken with an FV1000 confocal upright microscope system (Olympus) equipped with a 4× water-immersion lens (XLFluor, NA 0.28) and a 20× water-immersion lens (XLUMPlanFL, NA 1.0). The 405-nm, 473-nm, and 559-nm laser lines were used for the nuclear stain (Hoechst, DAPI), green fluorescence molecules (EGFP, Alexa488), and red fluorescence protein (mCherry), respectively. Images of mouse ductus arteriosus were obtained with an FV1000 equipped with a 60× oil-immersion lens (UPlanSApo, NA 1.35). Image files were processed and analyzed using FLUOVIEW Viewer software (Olympus) and Volocity (PerkinElmer). Images of the HUVECs were obtained with an inverted IX81 microscope with a 40× lens (UPlanSApo, NA 0.95) (Olympus), and analyzed using Metamorph (Molecular Devices).

The images of hyper-resolution microscopy were collected with an IX83 electric inverted microscope equipped with an SD-OSR device for hyper-resolution processing (Olympus), using a 100× silicon oil-immersion lens (UPLSAPO100xS).

### Chemical treatment and phalloidin stain of whole-mount zebrafish embryos

Zebrafish embryos were fixed with 4% paraformaldehyde (PFA) and permeabilized with 0.1% Triton-X in Phosphate Buffered Saline (PBS), and then incubated with Alexa488-conjugated phalloidin (Life technologies). Cell nuclei were stained with Hoechst 33342 (Invitrogen).

### Whole-mount *in situ* hybridization

Whole-mount *in situ* hybridization of zebrafish embryos was performed as described previously [34]. Pigmentation of embryos was inhibited with 0.04 mM 1-phenyl-2-thiourea (PTU) (Sigma-Aldrich) from 8 hpf.

### Sequence of morpholino and primers for zebrafish

The morpholino oligos we used for Tnnt2 (5’-CATGTTTGCTCTGATCTGACACGCA-3’) [35], zYap (5’-CTCTTCTTTCTATCCAACTGAAACC-3’) [36], and zTaz (5’-CTGGAGAGGATTACCGCTCATGGTC-3’) [37] were obtained from Gene Tools LLC. Zebra ribosomal protein L13a (zRPl13a) was detected with primers (forward: 5’- TCTGGAGGACTGTAAGAGGTATGC-3’, reverse: 5’- AGACGCACAATCTTGAGAGCA-3’) [38].

### Microinjection of plasmid or morpholino into zebrafish embryos

Plasmid DNA (100 ng/μl) mixed with injection buffer (120 mM KCl, 20mM Hepes, 0.25% phenol red) was microinjected into embryos at the one-cell stage. Morpholino oligos were also diluted with injection buffer to the described concentration.

### Cell culture and phalloidin staining

HEK293T cells were maintained in DMEM containing 10% FBS. HUVECs were cultured in EBM2 medium with additional supplements consisting of essential growth factors (Lonza, Switzerland). For phalloidin staining, Ctgf protein (Pepro Tech, USA) was added and the reaction was stopped using cooled PBS. Cells were stained with Alexa488-conjugated phalloidin (Life technologies) according to the manufacturer's protocol.

### Immunohistochemistry of mouse ductus arteriosus

Our experiments used P 0.5, P 0.75, and P 1.5 (C57BL/6 × DBA/2 strain; CLEA Japan, Tokyo, Japan). The ductus arteriosus and descending aortas were collected from the mice and fixed with 4% PFA, and then embedded in an optimal cutting temperature compound (SAKURA) for the production of frozen sections. After rinsing with PBS, they were then incubated with mouse anti-Yap monoclonal antibody (Abnova), which was detected using Alexa488-conjugated goat anti-mouse antibody (Life Technologies). Cell nuclei were stained with DAPI in a Vectashield Mounting Medium (VECTOR). The use of experimental animals was approved by the Animal Experiment Ethics Committees at the Institute of Molecular and Cellular Biosciences, University of Tokyo (#2714).

### Luciferase assay

HEK293T cells were lysed, processed, and assayed for luciferase activity using the Luciferase Assay System (Promega, USA), which was detected using a LAS-4000 (FujiFilm, Japan).

### Quantitative real-time RT-PCR

More than 10,000 endothelial cells of each fish line were collected by gating based on mCherry intensity, using a BD FACSAriaIII cell sorter. RNA extraction was performed using a Nucleospin RNA XS column (MACHEREY-NAGEL, Germany) and cDNA were synthesized with a Superscript III First-Strand Synthesis System primed with oligo (dT). The mRNA expression of hTead2ΔN was detected using a pre-designed Taqman probe with PrimeTime Mini qPCR Assay (Hs.PT. 58.4420176, IDT, USA). For hTead2ΔN, we performed a quantitative real-time PCR using a THUNDERBIRD Probe qPCR Mix (TOYOBO, Japan). As an internal control, zRPl13a were performed using a KAPA SYBR FAST Universal qPCR kit (KAPA Biosystems, USA).

### Quantification of circulation speed and blood pressure

To measure circulation speed, we obtained a continuous time-lapse image of blood cells expressing mCherry in the dorsal aorta (DA) using confocal microscopy FV1000 (Olympus), and analyzed according to the previous report [39].

To measure blood pressure, the embryo was mounted in 1% low-melting agarose on a 35 mm glass-bottomed dish. The dish was filled with fish medium, after which the cauda was cut with scissors and the medium was drained until body fluid seeped out from the cut edge. The height of the remaining medium (mm) was converted to blood pressure (mmHg).

### Statistical analysis

Data are expressed as mean ± SD. The statistical significance for paired samples was determined using Welch’s *t*-test.

## Results

### Yap/Taz transcriptional activity is required for caudal vein plexus formation during zebrafish angiogenesis

**Fig 1:**
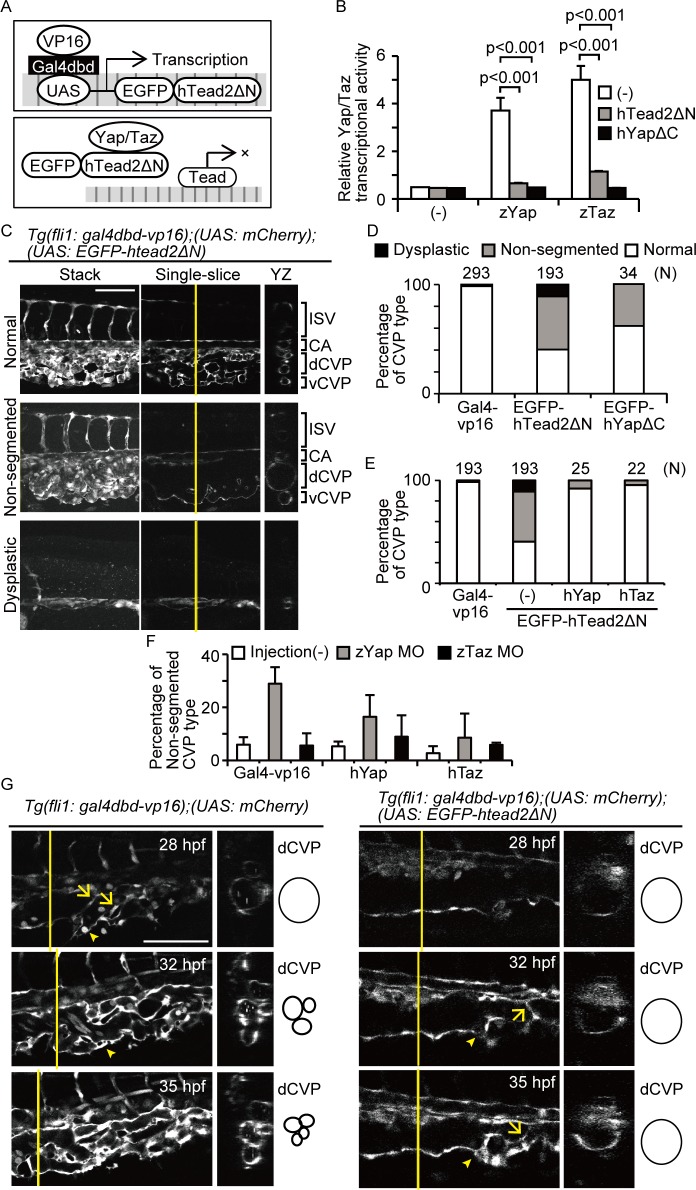
Yap/Taz transcriptional activity is essential for dCVP regression. (A) Schemas represent the model of dominant-negative expression. (B) HEK293T cells were transfected with pFR-Luc and pcDNA3.1-Gal4-hTead2ΔN. Cells were also introduced with p3xflag-cmv-14-zYap, -zTaz, pEGFP-hTead2ΔN, and -hYapΔC as indicated. Cells were harvested 24 hours after transfection and measured the luciferase activity. Data are means and SDs (*n* = 3). (C and D) Images of *Tg(fli1*: *gal4dbd-vp16);(UAS*: *mCherry);(UAS*: *EGFP-htead2ΔN)* at 35 hours post-fertilization (hpf) are shown. Cross-sectional images of the plane indicated by the yellow lines (right) also shown. White color represents mCherry signals. Scale bars: 100 μm. CA, caudal artery; dCVP, dorsal part of the CVP; vCVP, ventral part of the CVP; ISV, intersegmental vessel. The results including *Tg(fli1*: *gal4dbd-vp16);(UAS*: *EGFP-hyapΔC)* were quantitated and shown as (D). The result of *Tg(fli1*: *gal4dbd-vp16);(UAS*: *mCherry)* is shown as Gal4-vp16. (E) *Tg(fli1*: *gal4dbd-vp16);(UAS*: *EGFP-hTead2ΔN)* fish were crossed with *Tg(UAS*: *EGFP-hyap)* or *(UAS*: *EGFP-htaz)* and analyzed as in (D). (F) Embryos were injected with morpholino for zYap or zTaz. The percentage of fish with non-segmented CVPs at 31 hpf is shown. The numbers of embryos analyzed is at the top of each bar. The result of *Tg(fli1*: *gal4dbd-vp16);(UAS*: *mCherry)* is shown as Gal4-vp16. (G) Single-slice and cross-sectional images of indicated embryos are shown. Arrows indicate endothelial cells sprouting into the lumen. Arrowheads indicate vCVP formation. White color shows mCherry signals. Scale bars: 100 μm.

Yap knockout mice die in the first half of gestation as a result of vascular defects. Therefore, while the details are unclear, the activity of Yap can be considered essential for angiogenesis. To investigate how Yap/Taz transcriptional activity is essential for angiogenesis, we generated fish lines to specifically inhibit this activity in ECs. The *Tg(UAS*: *EGFP-htead2Δ)* line, which expresses a fusion protein of enhanced green fluorescent protein (EGFP) and human Tead2 delta N (hTead2ΔN), was coupled to an upstream activating sequence (UAS). The hTead2ΔN contains only the C-terminal Yap/Taz binding domain (amino acids 159–450), thus endogenous Yap/Taz failed to bind to DNA via transactivation (Fig 1A). We also established another fish line with EC-specific inhibition of Yap/Taz transcriptional activity by overexpressing a fusion protein, EGFP-hYapΔC, in ECs. The hYapΔC protein contains only the Tead binding domain (amino acids 47–154) and an abrogated transactivation domain, so endogenous Yap/Taz failed to bind to endogenous Tead.

To confirm whether hTead2ΔN and hYapΔC can inhibit Yap/Taz-Tead transcriptional activity, we examined a luciferase assay for detecting Yap-Tead or Taz-Tead transcriptional activity (Fig 1B). Relative luciferase activity increased in zYap- or zTaz-overexpressing cells compared with the control. Furthermore, co-expression of hTead2ΔN or hYapΔC abrogated zYap and zTaz transcriptional activity, supporting our hypothesis that hTead2ΔN and hYapΔC can inhibit Yap/Taz-Tead transcriptional activity.

We then crossed the *Tg(UAS*: *EGFP-htead2ΔN)* line with the *Tg(fli1*: *gal4dbd-vp16);(UAS*: *mCherry)* line, which expresses the Gal4 DNA binding domain (Gal4dbd) and VP16 (enhancer of transcriptional activity) under the control of the EC-specific fli1 promoter. The expression of mCherry indicates Gal4dbd expression. Compared with the control (*Tg(fli1*: *gal4dbd-vp16);(UAS*: *mCherr**y)*; Fig 1C, upper), we found that the dCVP expanded more in the *Tg(fli1*: *gal4dbd-vp16);(UAS*: *mCherry);(UAS*: *EGFP-htead2ΔN)* embryos at approximately 35 hours post-fertilization (hpf; Fig 1C, middle). We named this phenotype ‘non-segmented,’ based on the resulting morphology. There were also some embryos with defects in CVP formation, which we named ‘dysplastic’ (Fig 1C, bottom). Notably, in the formation of the intersegmental vessels (ISVs), there was no difference in development or duration between normal and non-segmented embryos, suggesting that Yap/Taz transcriptional activity is specifically required for caudal vein plexus formation. To quantify this effect (Fig 1D), 293 embryos of the control line and 193 embryos that expressing EGFP-hTead2ΔN were categorized, based on CVP formation, as normal, non-segmented, or dysplastic. Of the control embryos, only three were non-segmented, and the remaining 290 were normal. In contrast, in the EGFP-hTead2ΔN line, 94 embryos were non-segmented, 21 were dysplastic, and 78 were normal. These results suggest that Yap/Taz transcriptional activity is required for CVP formation. We also prepared *Tg(fli1*: *gal4dbd-vp16);(UAS*: *mCherry);(UAS*: *EGFP-hyapΔC)*, and observed CVP formation in this line. In the *Tg(UAS*: *EGFP-hyapΔC)* line, 13 (38.2%) embryos were non-segmented and 21 (61.8%) were normal. We thus concluded that Yap/Taz transcriptional activity is required for CVP formation.

To confirm our model, we tested whether overexpression of Yap or Taz in ECs can cancel the non-segmented CVP formation. We crossed the *Tg(fli1*: *gal4dbd-vp16);(UAS*: *mCherry);(UAS*: *EGFP-htead2ΔN)* line with a *Tg(UAS*: *EGFP-hyap)* or *Tg(UAS*: *EGFP-htaz)* line. We then counted the number of embryos with a non-segmented CVP at 31–32 hpf (Fig 1E). The number of embryos with a non-segmented CVP was smaller in the groups with overexpression of hYap or hTaz than in the group in which only EGFP-hTead2ΔN was overexpressed (shown as hTead2ΔN). These results support our hypothesis that hTead2ΔN inhibits Yap/Taz transactivation in the CVP.

To investigate further, we tried to determine which molecule between Yap and Taz is responsible for CVP formation, by crossing the *Tg(UAS*: *EGFP-hyap)* or *(UAS*: *EGFP-htaz)* line with *(fli1*: *gal4dbd-vp16)* and injecting morpholino (MO) for zYap or zTaz. We then counted the number of embryos with a non-segmented CVP at 31–32 hpf (Fig 1F). The percentage of such embryos was higher with Yap MO injection (29.0%) than in the control group (6.0%) (left two bars). However, there was no significant change as a result of Taz MO injection. Notably, the effect of Yap MO was partially canceled by overexpression of hYap (16.4%) or hTaz (8.6%). These results support that Yap is the key molecule responsible for CVP formation.

CVP formation is unique in angiogenesis because it involves both neogenesis and the convergence of circulation. To understand normal CVP formation, we monitored *Tg(fli1*: *gal4dbd-vp16);(UAS*: *mCherry)* embryos from 28 hpf to 35 hpf, which is when the most dynamic change in CVP remodeling occurs. First, the CVP was formed from one of the dorsal parts (dCVP). There was a one-way circulation of blood from the caudal aorta (CA) to the dCVP at 28 hpf (Fig 1G, upper left). Some ECs sprouted into the lumen of the dCVP for bridging (Fig 1G, indicated with arrows in the upper left image). At approximately the same time, the ECs migrated and started to form the ventral part of the CVP (vCVP), consisting of a new caudal vein under the dCVP (Fig 1G, indicated with an arrowhead in the left images). After the vCVP had formed and the blood flow had been divided, the dCVP began its segmentation into tiny veins through the bridging and migration of ECs (Fig 1G, middle left). This segmentation of the dCVP repeated, and the vCVP became the main circulatory vessels. Eventually, the dCVP lost blood flow at 35 hpf (Fig 1G, bottom left) via vessel shrinking, after which the vCVP converged to form the main caudal vein.

To investigate how the non-segmented dCVP phenotype is formed during these steps, we observed CVP formation in the *Tg(fli1*: *gal4dbd-vp16);(UAS*: *EGFP-htead2ΔN)* line. At 28 hpf, the CVP was formed alongside the dCVP, and was similar to the controls (Fig 1G, upper right). ECs then sprouted into the lumen of the dCVP for bridging (Fig 1G, indicated with an arrow in middle right image). Despite this, the segmentation of the dCVP was aborted at 35 hpf (Fig 1G, bottom right, ECs in the lumen indicated with an arrow). Notably, the sprouting of ECs to form the vCVP was similar to the control (Fig 1G, indicated with arrowheads in middle and bottom right images), suggesting that Yap/Taz activity plays a specific role in ECs that results in dCVP regression. These findings indicate that Yap/Taz transcriptional activity is required for dCVP segmentation during CVP formation.

### Yap/Taz transcriptional activity occurs in endothelial cells during dCVP segmentation

**Fig 2:**
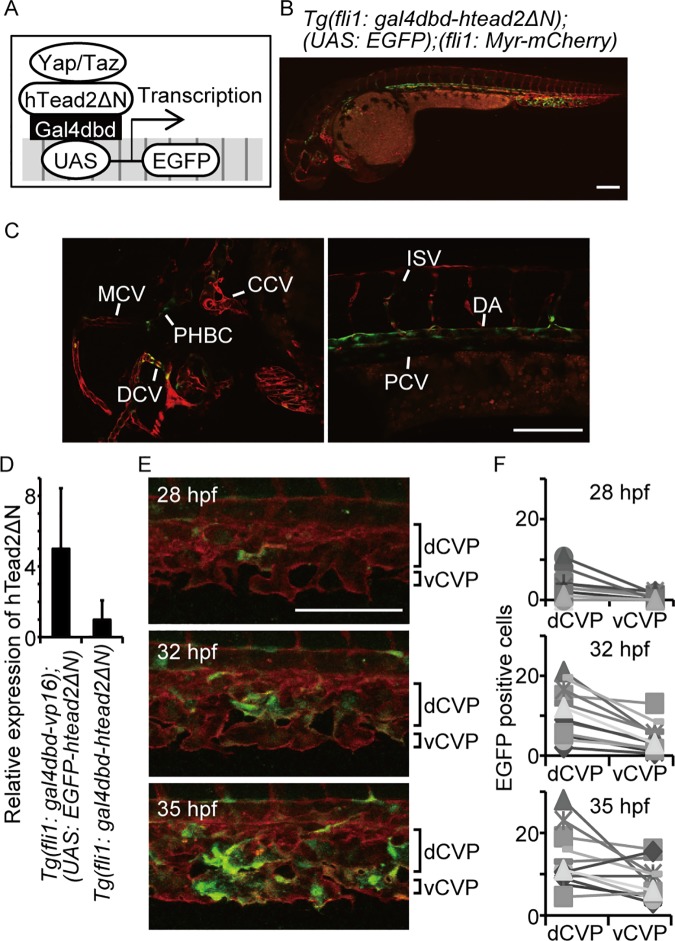
Yap/Taz is transactivated in the CVP during dCVP regression. (A) Schema for detecting Yap/Taz transcriptional activity is shown. (B and C) Confocal stack image of a *Tg(fli1*: *gal4dbd-htead2ΔN);(UAS*: *EGFP);(fli1*: *Myr-mCherry)* embryo at 36 hpf is shown. The EGFP signal is shown in green and mCherry in red. Scale bar: 100 μm. Enlarged images in (B) are also shown: head (left), dorsal area (right). CCV, common cardinal vein; PHBC, primordial hindbrain channel; DCV, dorsal ciliary vein; MCV, middle cerebral vein; DA, dorsal aorta; PCV, posterior cardinal vein. (D) The relative expression of hTead2ΔN mRNA in the indicated embryos are shown. Data are means and SDs (*n* = 3). (E and F) Representative images of the indicated embryo are shown. Scale bar: 100 μm. The numbers of EGFP-positive cells were counted in the dCVP and vCVP from 12 embryos.

We next endeavored to verify whether Yap/Taz transcriptional activity does indeed occur in ECs during CVP formation. For this purpose, we constructed a system for monitoring EC-specific Yap/Taz transcriptional activity by developing a transgenic zebrafish line expressing the fusion protein Gal4dbd-hTead2ΔN, under the control of the EC-specific fli1 promoter (Fig 2A). We crossed this line with *Tg(UAS*: *EGFP);(fli1*: *Myr-mCherry)* to visualize the activity of Yap/Taz in ECs specifically. These fish showed EGFP signals in the CVP, dorsal aorta, posterior caudal vein, primordial hindbrain channel, common cardinal vein, dorsal ciliary vein, and middle cerebral vein at 36 hpf (Fig 2B and 2C). These signals were all concurrent with the expression of the mCherry signal in the ECs. This indicates that Yap/Taz transcriptional activity increased in ECs during angiogenesis.

Although Tg expressed Gal4dbd-hTead2ΔN, which is a similar molecule to EGFP-htead2ΔN, most of the *Tg(fli1*: *gal4dbd-htead2ΔN);(UAS*: *EGFP)* embryos showed normal CVP formation. To understand this, we compared the expression levels of htead2ΔN between *Tg(fli1*: *gal4dbd-vp16);(UAS*: *EGFP-htead2ΔN)* and *Tg(fli1*: *gal4dbd-htead2ΔN);(UAS*: *EGFP)* embryos, using quantitative PCR methods (Fig 2D). Expression of htead2ΔN in *Tg(fli1*: *gal4dbd-htead2ΔN);(UAS*: *EGFP)* was one fifth of that in *Tg(fli1*: *gal4dbd-vp16);(UAS*: *EGFP-htead2ΔN)*, suggesting that the effect of Gal4dbd-hTead2ΔN on endogenous Yap/Taz-Tead transcriptional activity is trivial.

By monitoring fluorescence in the fish lines, we sought to identify the location and timing of Yap/Taz transcriptional activity during CVP formation. Initially, the EGFP signal was detected in the dCVP at approximately 28 hpf (Fig 2E, upper). At 32 hpf, the EGFP signal increased in both the dCVP and the vCVP (Fig 2E, middle) and remained positive until approximately 36 hpf (Fig 2E, bottom; S1 Movie). Interestingly, the numbers of EGFP positive cells were higher in dCVP than in vCVP during CVP formation (Fig 2F). We therefore concluded that Yap/Taz transcriptional activity increases in ECs during CVP formation, and initially increases in the dCVP, which then segments into thin veins.

### Yap/Taz transcriptional activity is induced by blood flow in the CVP

**Fig 3:**
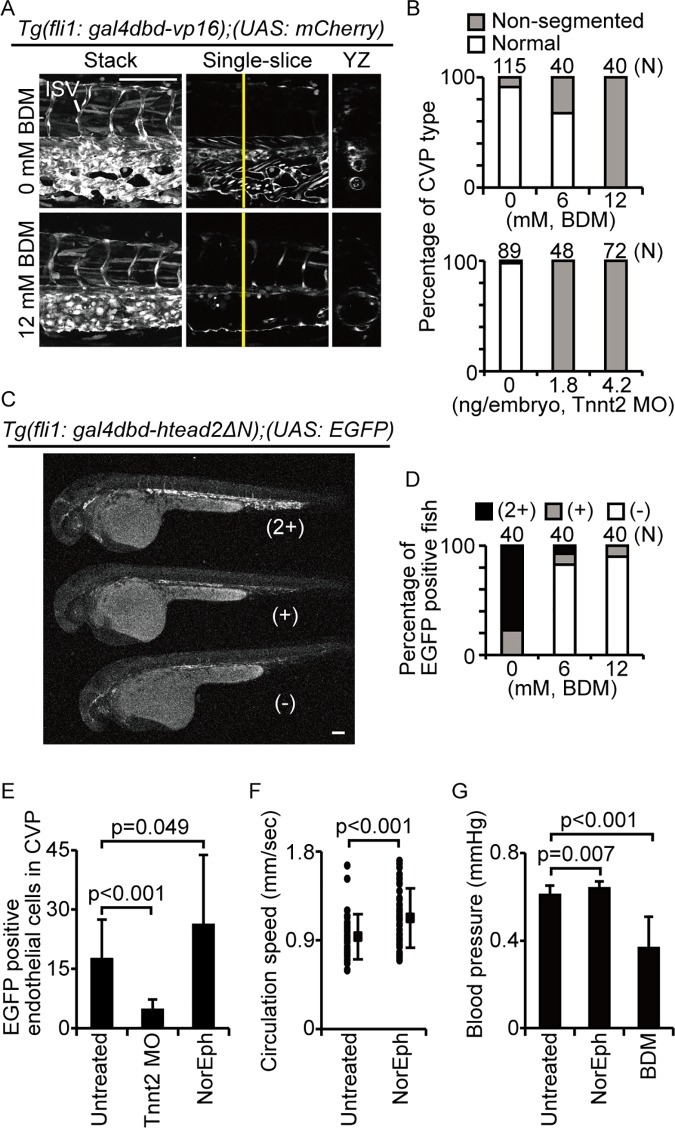
Yap/Taz is activated by blood flow in the CVP. (A and B) Images of mCherry intensity in indicated embryos at 36 hpf are shown. Embryos were treated with or without 12 mM BDM from 28–36 hpf and quantified. Scale bars: 100 μm Similar experiments were performed with morpholino for Tnnt2. (C and D) Representative images of EGFP signal levels in indicated embryos at 36 hpf are shown. Signal intensity was qualitatively categorized as low (−), intermediate (+), or high (2+), as shown. Scale bar: 100 μm. (E) The number of EGFP-positive cells in *Tg(fli1*: *gal4dbd-htead2ΔN);(UAS*: *EGFP)* embryo in CVP at 32 hpf are shown. Untreated embryos are shown as control (n = 14). Some embryos were treated with Tnnt2 MO (n = 18). Some embryos were treated with 1mM norepinephrine (NorEph) (n = 17). (F) Circulation speed of blood cells in fish treated with 1mM norepinephrine were measured as described materials and methods section. Each dot indicates the circulation speed of blood cells in one control embryo (*n* = 7) or norepinephrine-treated embryo (*n* = 10). Solid squares and error bars represent means and SDs. (G) Blood pressure in fish treated with 1mM norepinephrine or 12 mM BDM were measured. Data are means and SDs for the control (*n* = 24), norepinephrine treatment (*n* = 18), and BDM treatment (*n* = 7).

We then investigated the upstream factors of Yap/Taz transcriptional activity in the CVP. It has been reported that this activity is regulated by mechano-stress factors caused by the cellular microenvironment. We thus hypothesized that the circulation is the upstream factor inducing Yap/Taz transcriptional activity in the CVP, and investigated the relationship between circulation and CVP formation. We decreased circulation volume and blood pressure by treating the embryos with 2,3-Butanedione monoxime (BDM). BDM is an inhibitor of myosin ATPase and suppresses cardiac contraction. Relative to the control, BDM treatment induced the formation of a non-segmented CVP, similar to the dCVP phenotype of EGFP-hTead2ΔN (Fig 3A, single-slice and YZ images). Furthermore, in spite of the dCVP phenotype, the ISVs formed normally in BDM-treated fish (Fig 3A, stack images), implying that the effect of BDM treatment specifically disrupts the CVP formation. We treated 115, 40, and 40 embryos with 0, 6, and 12 mM BDM, respectively, and then counted the number of normal CVPs and non-segmented CVPs in each group (Fig 3B). These results suggest that blood flow is required for dCVP segmentation. To investigate this further, we used Tnnt2 morpholino (MO) to stop the heartbeat from the start (Fig 3B). We used two doses of injected Tnnt2 MO: 1.8 ng/embryo and 4.2 ng/embryo. Each group exhibited almost complete cardiac arrest. In both groups treated with Tnnt2 MO, all embryos had a non-segmented CVP, supporting that circulation is required for CVP formation.

We then investigated whether Yap/Taz-Tead transcriptional activity in the CVP is induced by circulation by treating *Tg(fli1*: *gal4dbd-htead2ΔN);(UAS*: *EGFP);(fli1*: *Myr-mCherry)* embryos with 6 mM or 12 mM BDM for 7 h (from 29 hpf to 36 hpf) and observing EGFP expression in the CVP (Fig 3C and 3D). The EGFP signal in treated embryos was lower than in control embryos. We categorized the embryos into three arbitrarily defined groups according to signal intensity: low (−), intermediate (+), and high (2+). The change in signal intensity was dependent upon the BDM concentration (Fig 3D), suggesting that circulation induces Yap/Taz-Tead transcriptional activity in the CVP.

We conducted similar experiments using Tnnt2 MO and norepinephrine (Fig 3E). Again, the absence of circulation, induced by Tnnt2 MO injection, reduced the number of EGFP-positive cells, whereas stronger circulation, induced by norepinephrine treatment, increased them. Importantly, the physiological status of the fish was consistent with the CVP phenotype (Fig 3F and 3G). We therefore concluded that Yap/Taz transcriptional activity in the CVP is triggered by circulation and is necessary for dCVP regression.

### Yap/Taz transcriptional activity controls vessel shrinking during dCVP segmentation

**Fig 4:**
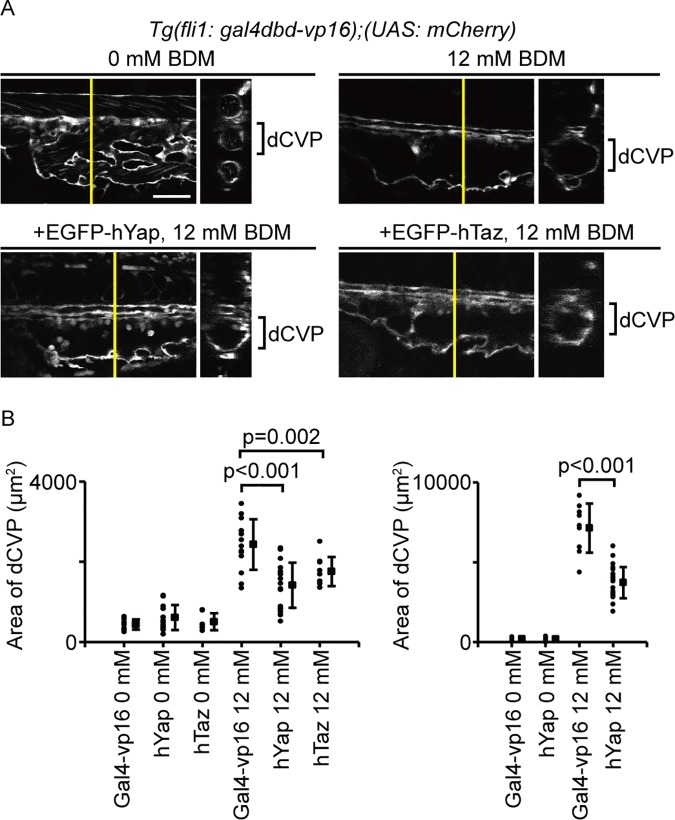
Yap/Taz transcriptional activity controls vascular shrinking during dCVP regression. (A) Embryos were treated with or without 12 mM BDM from 28–36 hpf. Lateral single-slice images of the CVP region in indicated embryos at 36 hpf are shown. Cross-sectional images in the plane indicated by the yellow lines are shown to the right. White color shows mCherry signals. Scale bars: 100 μm. (B) Embryos were treated with or without 12 mM BDM from 28–36 hpf and 28–48 hpf, respectively. Each dot represents one embryo, and solid squares and error bars represent means and SDs.

We next investigated how Yap/Taz transcriptional activity controls dCVP segmentation by developing *Tg(UAS*: *EGFP-hyap)* and *Tg(UAS*: *EGFP-htaz)* lines. Fish were crossed with *Tg(fli1*: *gal4dbd-vp16)* to obtain embryos that overexpressed Yap or Taz in their ECs. We then treated the embryos with 0 mM BDM (control; Fig 4A, upper left) or 12 mM BDM (Fig 4A, upper right). Both the Yap-overexpressing (Fig 4A, bottom left) and the Taz-overexpressing fish (Fig 4A, bottom right; Fig 4B, left) had narrower dCVPs than the control embryos at 36 hpf. Furthermore, the difference between Yap-overexpressing zebrafish and the control embryos was greater at 48 hpf (Fig 4B, right). Together, these results suggest Yap/Taz transcriptional activity plays a role in shrinking vessels during dCVP segmentation.

### Ctgf expression in the CVP is induced by Yap/Taz transcriptional activity and is required for dCVP regression

**Fig 5:**
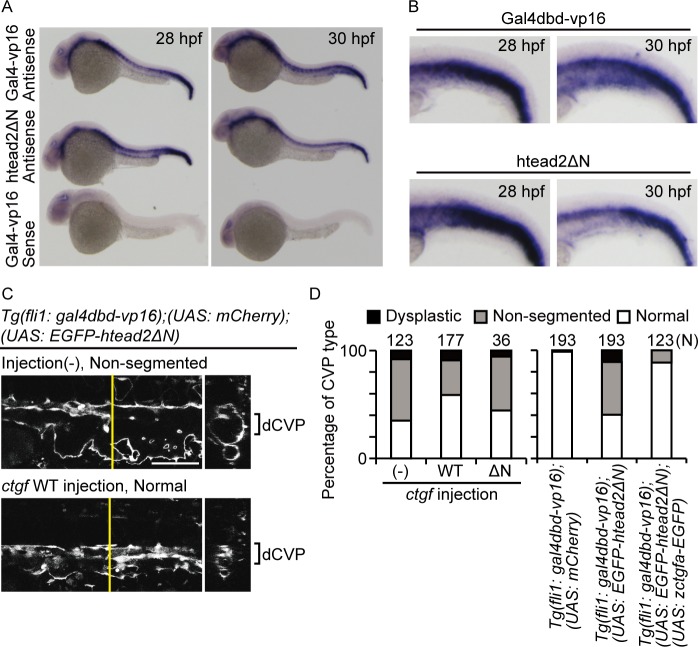
Ctgf expression depends on Yap/Taz transcriptional activity in the CVP and is required for dCVP regression. (A and B) Expression patterns of *ctgf* mRNA at 28 and 30 hpf are shown. Embryos of *Tg(fli1*: *gal4dbd-vp16);(UAS*: *EGFP-hTead2ΔN)* and *Tg(fli1*: *gal4dbd-vp16)* are indicated as hTeadΔN and Gal4-vp16, respectively. A sense probe was used to confirm the specificity of the antisense probe reaction. Enlarged images in (A) are also shown. (C and D) Lateral single-slice images of the CVP region at 36 hpf are shown. Injection (-): embryos not injected; ctgf WT: injected with UAS-zctgfa-EGFP. White color shows mCherry signals. Scale bar: 100 μm. Quantified results are also shown.

Although Yap/Taz transcriptional activity was observed in a portion of the CVP cells, the non-segmented phenotype was the result of a defect in the segmentation of the whole dCVP. Given these results, we hypothesized that a downstream factor of Yap/Taz might affect other ECs, via secretion. Connective tissue growth factor (Ctgf) is a secretion protein and one of the target molecules of Yap. Thus, using whole-mount *in situ* hybridization, we investigated whether *ctgf* mRNA expression was controlled by Yap/Taz transcriptional activity in the CVP (Fig 5A). In control fish, we detected *ctgf* mRNA expression in the CVP from 30 hpf (Fig 5B, upper), but failed to detect it at 28 hpf. Meanwhile, the fish expressing EGFP-hTead2ΔN showed a lower signal than the control fish (Fig 5B, bottom right). These results indicate that the expression of *ctgf* mRNA is positively regulated by Yap/Taz transcriptional activity during dCVP segmentation. To confirm whether Ctgf expression regulates dCVP closure, we overexpressed Ctgf in ECs and investigated the effect of Ctgf expression during dCVP regression. First, we prepared *Tg(fli1*: *gal4dbd-vp16);(UAS*: *EGFP-htead2ΔN)* embryos microinjected with plasmids UAS-zctgfa-EGFP (WT) or UAS-zctgfaΔN-EGFP (ΔN). The sequence *zctgfaΔN* lacks its N-terminal for secretion, so it works as a control for Ctgf overexpression in the CVP. We observed CVP formation at 36 hpf, and categorized the three types of embryos (control, WT, and ΔN) according to CVP formation as follows: normal, non-segmented, or dysplastic (Fig 5C and 5D). In the control group, 56.9% of embryos had a non-segmented CVP, whereas. WT plasmid injection resulted in 32.2% of the embryos having a non-segmented CVP (Fig 5C, lower). The proportion of embryos with a dysplastic CVP in the control, WT, and ΔN groups was 8.1%, 9.0%, 5.6%, respectively, and there were no significant differences between the groups (Fig 5D). We conducted similar experiments using *Tg(fli1*: *gal4dbd-vp16);(UAS*: *EGFP-htead2ΔN);(UAS*: *zctgfa-EGFP)*. The forced expression of zCtgf in this line abrogated the non-segmented phenotype. These results indicate that Ctgf expression in ECs is involved in dCVP regression and is a key target of Yap/Taz during this process.

### Yap/Taz activity increases F-actin in ECs during CVP formation

**Fig 6:**
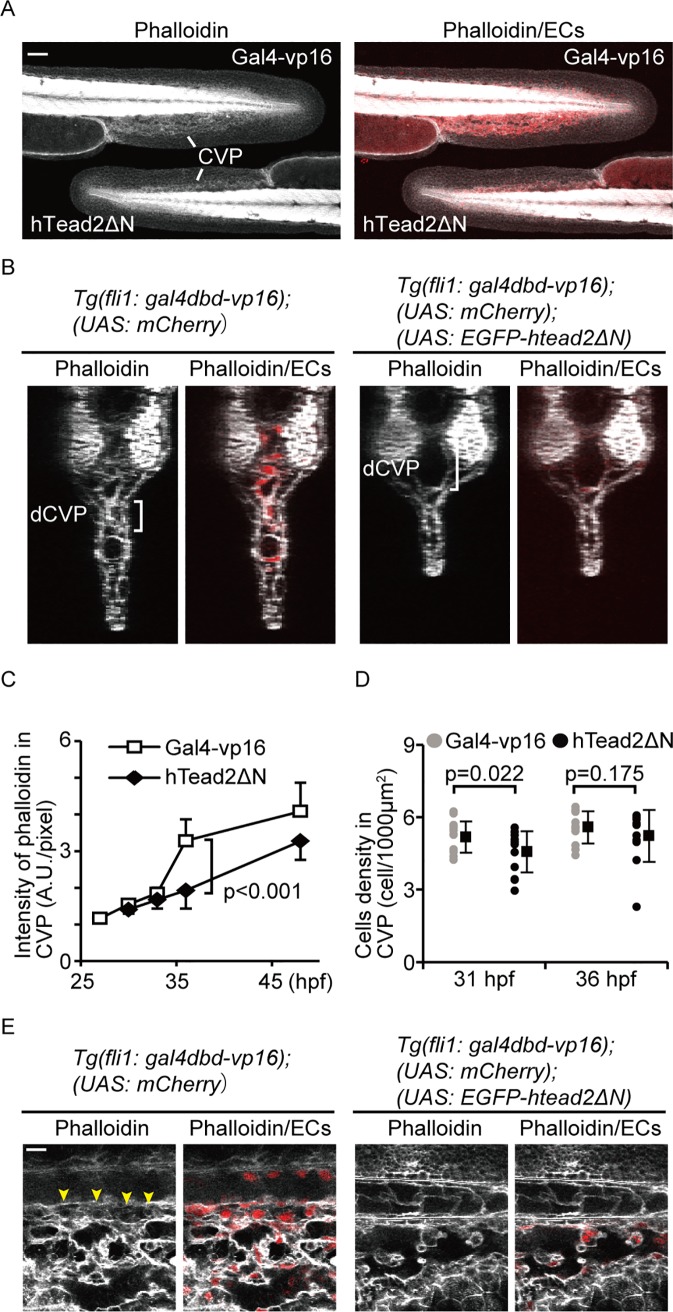
Actin polymerization is induced by Yap/Taz transcriptional activity in the CVP. (A, B, and C) Images of a phalloidin stain of indicated embryos at 36 hpf are shown. Embryos of *Tg(fli1*: *gal4dbd-vp16);(UAS*: *EGFP-hTead2ΔN);(UAS*: *mCherry)* and *Tg(fli1*: *gal4dbd-vp16);(UAS*: *mCherry)* are indicated as hTeadΔN and Gal4-vp16, respectively. White represents the phalloidin stain. Red indicates mCherry signals. Scale bars: 50 μm. Time-dependent change in the phalloidin stain during CVP formation were also analyzed and shown in (C). Solid squares and error bars represent means and SDs. (D) Cell densities of endothelial cells forming the CVP in embryos are shown. Each dot represents one embryo, and solid squares and error bars represent means and SDs. (E) Enlarged images of (A) are shown. Scale bar: 10 μm.

Our results above suggest that Yap/Taz transcriptional activity controls cell structure by inducing Ctgf expression. Furthermore, Ctgf has been reported as a promoting factor for F-actin formation [40, 41]. We thus hypothesized that Yap/Taz transcriptional activity is required for controlling F-actin formation, to maintain cell structure during dCVP segmentation. To test this hypothesis, we attempted to detect F-actin using Alexa488-conjugated phalloidin in the zebrafish CVP. As a control, we stained *Tg(fli1*: *gal4dbd-vp16);(UAS*: *mCherry)* embryos at 27 hpf, 31 hpf, 36 hpf, and 48 hpf (Fig 6A, 6B and 6C). The signal intensity increased from 31 hpf until 48 hpf (Fig 6C). The greatest change occurred between 31 hpf and 36 hpf, which is also when the dCVP segmented into thin vessels. In contrast, the intensity of the signal in *Tg(fli1*: *gal4dbd-vp16);(UAS*: *EGFP-htead2ΔN)* embryos at 36 hpf did not increase relative to the control embryos, implying that Yap/Taz upregulates F-actin formation in ECs. Notably, the cell density of ECs in the CVP was comparable between the control embryos and those expressing hTead2ΔN at 36 hpf (Fig 6D), suggesting the higher phalloidin intensity is caused by induced actin polymerization but not cell density. From these results, we conclude that F-actin polymerization is induced by Yap/Taz transcriptional activity in the CVP.

We then investigated the localization of F-actin in the dCVP. F-actin structure regulates different aspects of cell behavior, including cell migration, cell-cell adhesion, and cell stiffness. To elucidate the role of F-actin structure during dCVP segmentation, we observed dCVPs in phalloidin-stained *Tg(fli1*: *gal4dbd-vp16);(UAS*: *mCherry)* embryos. The image shows that F-actin structure was enriched in the cytoplasm in regressed vessels specifically (Fig 6E, arrowheads). It has been reported that cytoplasmic and cell membrane F-actin are essential for cell stiffness, especially for lamellipodia protrusion [42] and erythrocyte shape maintenance [43], respectively. Our results suggest that Yap/Taz-dependent F-actin formation generates a mechanical force that may maintain cell stiffness.

### Actin polymerization is required for dCVP regression

**Fig 7:**
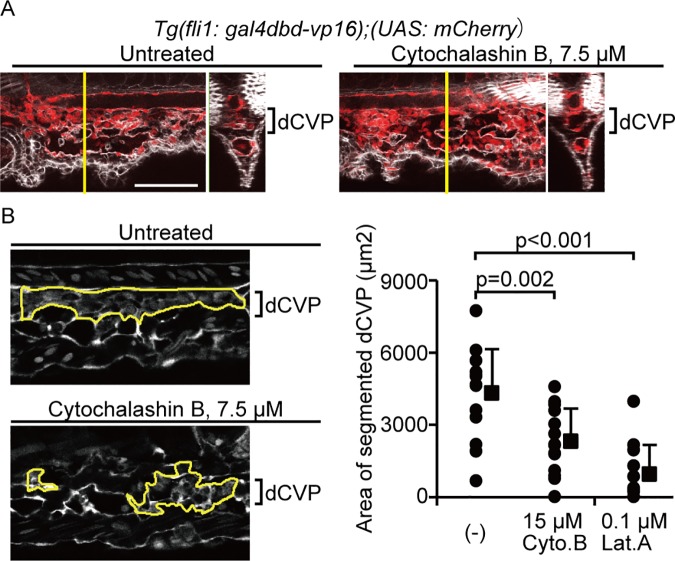
Actin polymerization is required for dCVP regression. (A) Image of phalloidin stain (white) merged with the mCherry signal (red) in indicated embryos treated with 0 μM or 7.5 μM Cytochalashin B from 30–37 are shown. Scale bars: 100 μm. (B) Representative images for gating of the segmented dCVP area in embryos are shown. The *Tg(fli1*: *gal4dbd-vp16);(UAS*: *mCherry)* embryos treated with 15 μM Cytochalasin B (Cyto.B) or 0.1 μM Latrunculin A (Lat.A) from 30–37 hpf. White color shows mCherry signals. Scale bars: 50 μm. The area of segmented dCVP, gated as indicated in (B) were quantified. The numbers of embryos analyzed are shown at the top of each bar. Each dot represents one embryo, and solid squares and error bars indicate means and SDs.

Next, we investigated whether F-actin formation is involved in dCVP regression by treating *Tg(fli1*: *gal4dbd-vp16);(UAS*: *mCherry)* embryos with 7.5 or 15 μM Cytochalasin B, or 0.1 μM Latrunculin A. These chemical reagents are known to be actin polymerization inhibitors. Since actin polymerization is essential for vCVP sprouting and its formation [44], we treated embryos with F-actin inhibitors just after vCVP formation. When the control (untreated) embryos showed segmented dCVPs for regression (Fig 7A), the embryos treated with 7.5 μM Cytochalasin B showed non-segmented dCVPs. These results suggest that F-actin formation is required for dCVP regression. To confirm this, we measured the segmented area in the dCVP (Fig 7B). The segmented area was significantly reduced by inhibiting actin polymerization, supporting that actin polymerization is required for dCVP segmentation and regression.

### F-actin induced by Ctgf shows a mesh-like structure in HUVECs

**Fig 8:**
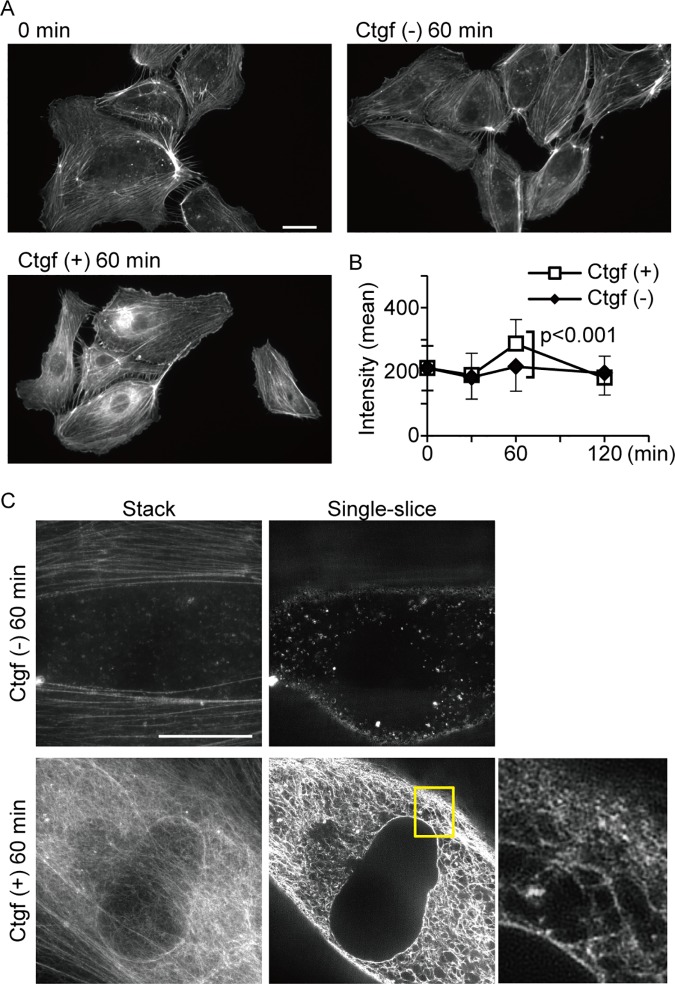
Actin polymerization is enhanced in HUVECs exposed to Ctgf protein. (A and B) Representative images of F-actin structure in HUVECs are shown in (A). Cells were exposed to Ctgf protein at the indicated times and stained with phalloidin. The phalloidin signal is shown in white. Phalloidin signal intensities in the perinuclear region of HUVECs were also quantified. Each square and error bar represents mean and SD calculated from more than 74 cells at each time point. (C) Confocal images of the phalloidin signal were obtained by hyper-resolution microscopy. Enlarged image of boxed area is shown. Scale bars: 10 μm.

We thus demonstrated that F-actin increases in ECs during dCVP segmentation, under the control of Yap/Taz transcriptional activity. Furthermore, Yap/Taz activity also induces *ctgf* mRNA. We therefore examined whether actin polymerization is induced by Ctgf protein *in vitro*. Human umbilical vein endothelial cells (HUVECs) were treated with 10 ng/ml Ctgf, and F-actin structures were detected with 488-phalloidin. The perinuclear area for cells treated with Ctgf increased relative to untreated cells (Fig 8A and 8B). This shows that Ctgf induces actin re-organization within approximately one hour.

To elucidate the structure of F-actin induced by Ctgf, we visualized the cells using hyper-resolution microscopy. A mesh-like structure was observed in the perinuclear area (Fig 8C), which connected the nuclear membrane and cellular membrane. These structures are highly consistent with our model of induced vascular regression, whereby the Yap/Taz-Ctgf pathway generates a mechanical force via actin polymerization.

### Yap/Taz transcriptional activity is involved in the closure of mouse ductus arteriosus

**Fig 9:**
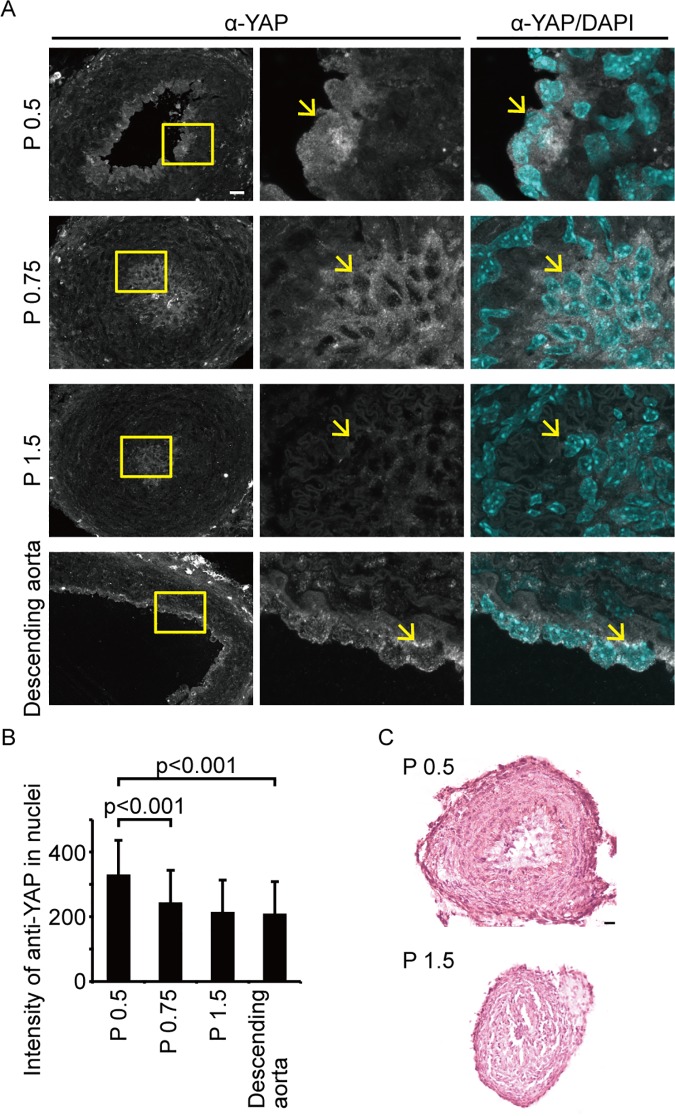
YAP nuclear accumulation in endothelial cells occurs at the initiation of mouse ductus arteriosus closure. (A) Confocal images of YAP localization in mouse vessels are shown. The top three rows show sections of the ductus arteriosus at post natal day (P) 0.5, P 0.75, and P 1.5, respectively. The bottom row shows a section of the descending aorta at P 1.5. White staining is the Alexa488 signal, indicating anti-YAP antibody. Blue staining shows nuclei detected by the DAPI stain. The boxed areas in the left column are enlarged in the two right columns. Arrows indicate endothelial cell nuclei in the lumen. Scale bars: 20 μm. (B) Intensity of the anti-YAP signal in cell nuclei were analyzed. The data are obtained from two independent experiments. Data are means and SDs. (C) HE stains of the ductus arteriosus at P 0.5 (left) and P 1.5 (right). Scale bars: 25 μm.

In our experiments, Yap/Taz transcriptional activity was required for vessel shrinking and dCVP regression. To confirm the involvement of YAP/TAZ activity during vascular regression in mammalian species, we tested another vessel: the ductus arteriosus of mice. It has reported that the closure of the ductus arteriosus occurs within one day of delivery, via infilling with the ECs [15]. We investigated the localization of YAP in ECs because YAP transcriptional activity is well correlated with its nuclear localization (Fig 9). In post natal day (P) 0.5 mice with an opened ductus arteriosus, YAP was distributed throughout both the nucleus and the cytoplasm, implying that it is activated during this stage (Fig 9A, upper). By P 0.75, the YAP signal had decreased in the ECs, specifically in the nuclear area (Fig 9A, second from top). However, there was a strong total YAP signal in the descending aorta at P 1.5 (Fig 9A, bottom), and in the ductus arteriosus at P 0.5 in the ECs. However, the signal intensity in the nuclear area of the descending aorta was as low as the ductus arteriosus signal at P 1.5, when the ductus arteriosus closure had already occurred (Fig 9B). We also observed the ECs existing inside the lumen with HE stain (Fig 9C) as reported previously [15]. These results show that YAP localizes in the nuclear area when ductus arteriosus closure is initiated, and translocates to the cytoplasm once closure is complete. Furthermore, this YAP localization is specific to ductus arteriosus ECs. Collectivity, these data support our hypothesis that Yap/Taz is activated during vascular regression.

## Discussion

In this paper, we identified the role of Yap/Taz transcriptional activity in ECs during zebrafish angiogenesis. The Yap/Taz transcriptional activity was upregulated by circulation, and affected vascular regression in the dCVP. Yap/Taz transcriptional activity regulated vascular size and induced shrinking via Ctgf expression and actin polymerization. Our results demonstrated that blood flow enhances vascular closure by Yap/Taz.

It has been reported that EC-specific Yap/Taz transcriptional activity is enhanced by mechanical stress [21, 22, 45]. However, the link between phenotypical changes and Yap/Taz activity are poorly understood. The difficulty in understanding the role of Yap/Taz in ECs might result from several issues, including the variety of ECs, changes in the circulation during development caused by vascular remodeling, and interactions with other, non-EC tissues. To overcome these problems, we established a system to inhibit Yap/Taz transcriptional activity in zebrafish ECs. This system is also a useful tool for understanding the role of Yap/Taz in other tissues because other promoters can easily be utilized.

From our observations, it seems that disturbances or changes in flow might be a cue for Yap/Taz activation. Previous investigations have shown that the response of ECs to laminar flow is different to turbulent flow [46–48]. In our observation, the cells expressing EGFP in the Yap/Taz monitoring fish were of the upper sides of both the dorsal aorta and the posterior caudal vein where tip cells sprouted for ISV formation. During CVP formation, the EGFP signals were detected where cells were sprouting and forming the vCVP (Fig 2E and 2F). Furthermore, Yap was localized in the nucleus when the blood flow changes direction from aortic arch to pulmonary artery in ductus arteriosus after birth. Based on this evidence, we propose a model whereby Yap/Taz transcriptional activity in ECs occurs when there is an alteration in blood flow.

Shear stress derived from the blood flow is considered to be a key factor for vascular regression, especially in the mouse yolk sac [49] and during the closure of the ductus arteriosus [50]. In this study, we identified that Yap/Taz transcriptional activity acts as a mechanosensor of blood flow to regulate vascular shrinking for vascular regression. The loss of Yap causes abnormal yolk sac angiogenesis [24], and our results could answer why this defect is specific to yolk sac in Yap knockout mice. In our results, vascular regression in the dCVP has at least three steps, endothelial cell bridging, vascular segmentation, and vascular shrinking. Yap/Taz transcriptional activity is only involved in vascular shrinking under BDM treatment. Hence, there are other mechanisms involved in the blood flow-induced regression of vessels. Integrin and angiotensin II have been reported as a mechanosensor and a mechanoregulator, respectively [51–53], and it has been suggested that they are both key factors in ductus arteriosus closure [54, 55], that may also be involved in dCVP regression.

In addition to the CVP, we detected EGFP signal in the dorsal aorta, posterior caudal vein, primordial hindbrain channel, common cardinal vein, dorsal ciliary vein, and middle cerebral vein by using Yap/Taz monitoring fish (Fig 2C). However, abnormalities during angiogenesis due to the inhibition or activation of Yap/Taz were not pronounced. Most likely, this is a limitation of our monitoring system. The EGFP signal indicates only the localization of Yap/Taz in the nucleus. Indeed, *ctgf* mRNA expression, which is considered the major downstream target of Yap/Taz transcriptional activity, was not detected in the dorsal aorta or posterior caudal vein, whereas the EGFP signal was observed in both using our monitoring system. Yap/Taz transcriptional activity occurs when it binds to Tead [56], and Yap/Taz is additionally regulated by other factors such as Smad, Runx2, and p73 [57–59]. Our monitoring system may not reflect the functional activities of Yap regulated by those factors. The unknown roles of Yap/Taz transcriptional activity in ECs may be uncovered by using another system that can detect differences in its functional activity *in vivo*. Other possibility is that Yap/Taz transcriptional activity in ECs is required for tissue genesis besides angiogenesis. Several studies have been shown that vascular networks promote osteogenesis [60, 61], neurogenesis [62, 63], and differentiations of hematopoietic stem cells [64, 65]. Indeed, CVP in zebrafish perform as a hematopoietic function. It will be worth to investigate the impact of Yap/Taz transcriptional activity in ECs to other tissues.

## Supporting information

S1 Movie In ECs, Yap/Taz is activated during CVP formation. Time-lapse video of CVP formation of *Tg(fli1*:*gal4dbd-hTead2ΔN);(UAS*:*EGFP);(fli1*:*Myr-mCherry)* from 28.5 hpf to 36 hpf. Green shows the EGFP signal and red shows the mCherry signal. The merging of the EGFP signal with the mCherry signal was first detected in part of the dCVP at 28.5 hpf. The EGFP signal increased until 36 hpf in both the dCVP and vCVP.
(WMV)
